# Metabolic Profiling and Identification of Shikonins in Root Periderm of Two Invasive *Echium* spp. Weeds in Australia

**DOI:** 10.3390/molecules22020330

**Published:** 2017-02-21

**Authors:** Dominik Skoneczny, Paul A. Weston, Xiaocheng Zhu, Geoff M. Gurr, Ragan M. Callaway, Russel A. Barrow, Leslie A. Weston

**Affiliations:** 1 Graham Centre for Agricultural Innovation, Charles Sturt University, Wagga Wagga, NSW 2678, Australia; pweston@csu.edu.au (P.A.W.); xzhu@csu.edu.au (X.Z.); ggurr@csu.edu.au (G.M.G.); leweston@csu.edu.au (L.A.W.); 2 Institute of Applied Ecology, Fujian Agriculture & Forestry University, Fuzhou 350002, China; 3 Division of Biological Science, University of Montana, 32 Campus Dr, Missoula, MT 59812, USA; ray.callaway@mso.umt.edu; 4 Research School of Chemistry, Australian National University, Acton, ACT 2601, Australia; rab@anu.edu.au

**Keywords:** metabolomics, UHPLC MS Q-TOF, plant defence, secondary plant products, naphthoquinones, rhizosphere

## Abstract

Metabolic profiling can be successfully implemented to analyse a living system’s response to environmental conditions by providing critical information on an organism’s physiological state at a particular point in time and allowing for both quantitative and qualitative assessment of a specific subset(s) of key metabolites. Shikonins are highly reactive chemicals that affect various cell signalling pathways and possess antifungal, antibacterial and allelopathic activity. Based on previous bioassay results, bioactive shikonins, are likely to play important roles in the regulation of rhizosphere interactions with neighbouring plants, microbes and herbivores. An effective platform allowing for rapid identification and accurate profiling of numerous structurally similar, difficult-to-separate bioactive isohexenylnaphthazarins (shikonins) was developed using UHPLC Q-TOF MS. Root periderm tissues of the invasive Australian weeds *Echium plantagineum* and its congener *E. vulgare* were extracted overnight in ethanol for shikonin profiling. Shikonin production was evaluated at seedling, rosette and flowering stages. Five populations of each species were compared for qualitative and quantitative differences in shikonin formation. Each species showed little populational variation in qualitative shikonin production; however, shikonin was considerably low in one population of *E. plantagineum* from Western New South Wales*.* Seedlings of all populations produced the bioactive metabolite acetylshikonin and production was upregulated over time. Mature plants of both species produced significantly higher total levels of shikonins and isovalerylshikonin > dimethylacrylshikonin > shikonin > acetylshikonin in mature *E. plantagineum*. Although qualitative metabolic profiles in both *Echium* spp. were nearly identical, shikonin abundance in mature plant periderm was approximately 2.5 times higher in perennial *E. vulgare* extracts in comparison to those of the annual *E. plantagineum.* These findings contribute to our understanding of the biosynthesis of shikonins in roots of two related invasive plants and their expression in relation to plant phenological stage.

## 1. Introduction

A plant’s metabolome is a reflection of its physiological state at a particular point in time under given conditions and can be successfully studied using a systematic approach, more commonly referred to as metabolomics [1,2,3]. In contrast, metabolic profiling is a focused approach that allows an in-depth analysis of selected metabolites, while successfully minimizing matrix complexity [3]. Metabolic profiling typically focuses on compounds of similar origin or similar chemical properties, and is often useful in defining biosynthetic pathways, joint metabolic networks or organismal responses to environmental changes [4]. Metabolic profiling can be performed using both targeted and untargeted approaches to evaluate most abundant metabolites present in a particular system. Qualitative and quantitative approaches to metabolic profiling can provide information about an organism’s response to varying environmental conditions, regulation of biosynthetic pathways and impact of genotype on metabolite expression [5,6]. Recent research has also demonstrated the suitability of metabolic profiling for investigation of biosynthetic pathways of plant secondary products [3,7,8,9] and also as an aid in the direct comparison of chemical profiles of various plant species [10].

Ultra-high pressure liquid chromatography coupled to quadrupole/time of flight mass spectrometry (UHPLC Q-TOF MS) is a powerful tool for separation and identification of secondary plant products. It enables identification of compounds by their accurate mass and typically generates structural information for compounds of interest in terms of unique fragmentation patterns or retention times specific to certain related compounds or groups of compounds [11]. Identification of metabolites using UHPLC Q-TOF data is facilitated by the comparison of sample results with those of compound databases and libraries for structural comparison. However, the majority of library-based metabolic structural data is based on results generated from model plant systems of *Oryza* and *Arabidopsis;* therefore studying novel or unknown plant metabolites remains a challenging task [12,13,14].

Plant secondary products are considered nonessential to organismal function; however, they are of critical importance in the regulation of trophic interactions, plant defence or adaptation to climatic conditions [15,16]. Numerous researchers have studied plant secondary products in foliar tissues, but many secondary metabolites derived from plant roots and the soil rhizosphere remain under-investigated [3]. Roots and root exudates often contain high concentrations of secondary products which are either actively or passively exuded, or released directly into rhizosphere where they interact with insects and herbivores, microbiota and/or other plants [17,18]. Plant exudates or root-released secondary products are typically represented by a diverse range of organic chemicals, and are often localized in specialized root tissues or organs in sufficient concentrations to act as phytotoxins [16,19]. Once in the rhizosphere, these compounds can act as microbial signalling molecules or antibiotics, thereby impacting seed germination or influencing plant growth and development [12,20,21]. In some cases, plant secondary products also influence invasive species success through their adverse impacts on native plants or microbial communities [12,22]. In the case of *Echium plantagineum* and *E. vulgare*, two successful invaders in Australia, bioactive secondary products, the shikonins could be associated with the success of these species in their novel range through their ability to influence rhizosphere interactions in the root zone where they are released from living roothairs and periderm of the *Echium* taproot. Other members of the Boraginaceae are also known to produce isohexenylnapthazarins with strong bioactivity as antimicrobials, insecticides and allelochemicals [23,24,25,26].

This study aimed to evaluate bioactive secondary products including shikonins that are concentrated in the root periderm of *E. plantagineum* and *E. vulgare*. In their native range of the Iberian Peninsula, these species are not common [27,28] and are often found in diverse mixtures of pasture grasses and herbaceous species. However, since their introduction to Australia in the 1800’s, they have become highly successful invaders. *Echium plantagineum*, also known as Paterson’s curse, is an annual weed now naturalized over 30 M ha where it dominates roadside plant communities and pastures [29,30]. In contrast, *E. vulgare*, or viper’s bugloss*,* is perennial and is limited to areas of higher rainfall and elevation in Southern Australia [27,31]. Both species were introduced to Australia either as ornamental pot plants [30] or more likely as contaminants of hay associated with importation of merino sheep [32,33]. Much less abundant across Southern Australia than Paterson’s curse, the perennial *E. vulgare* does not have major impacts on Australian agriculture [27]. However, the prevalence and toxicity of *E. plantagineum* causes significant impacts on Australia’s textile and meat industries, causing an estimated annual loss of AUD $250 M and reduced pasture quality [34]. Despite the negative effects of *E. plantagineum* on invaded ecosystems and its hepatotoxicity to grazing animals, the chemical composition of its belowground root system and potential impacts on rhizosphere interactions have been poorly explored.

Numerous *Echium* and other boraginaceous spp. accumulate bioactive secondary products including pyrrolizidine alkaloids mainly in foliar tissues [9,26,35] and isohexenylnaphthazarins (otherwise known as naphthoquinones or more specifically as shikonins) in the periderm of the roots [8,24,26]. In contrast, the interior of the root, or root cortex, does not produce shikonins [33,36]. Alkannins and shikonins (structural enantiomers) are lipophilic red pigments that accumulate in the periderm of certain Boraginaceae used as medicinal plants for their purgative, antimicrobial and wound-healing properties [24,37,38,39,40].

Interestingly, shikonins are derived from both the phenylpropanoid and isoprenoid biosynthetic pathways, both of which are typically upregulated under stress conditions [23]. Some of the key initial steps in isohexenylnaphthazarin biosynthesis have been revealed through identification of enzymes regulating biosynthesis and precursors including deoxyshikonin, the proposed precursor of shikonin [24]. Subsequent steps in the biosynthesis of shikonin derivatives remain unidentified and the pathway itself may vary among species [24].

**Figure 1:**
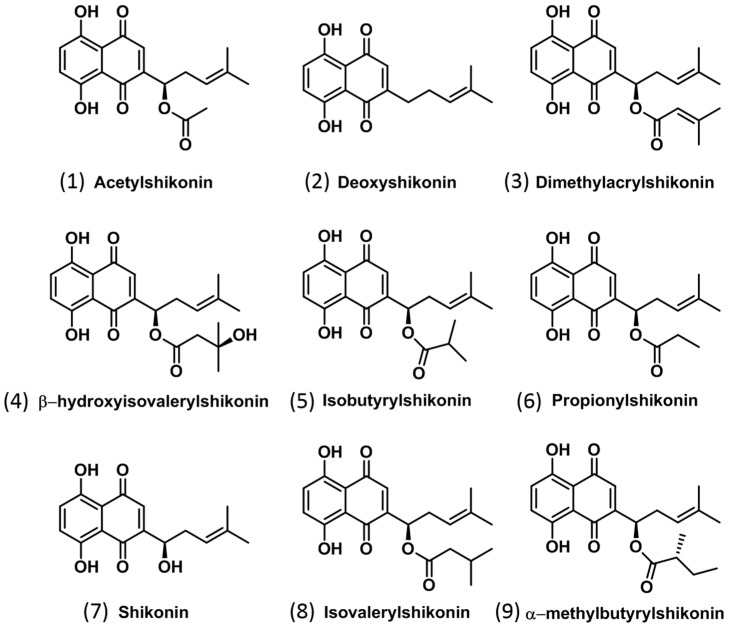
Structures of shikonins identified in *Echium plantagineum* and *E. vulgare* (Table 1). Isolvaleryshikonin and α-methylbutyrylshikonin are referred to as compound **8** and compound **9**, respectively.

Isohexenylnaphthazarins in general, and more specifically shikonins, are highly reactive chemicals, interacting with plant, microbial and mammalian systems and affecting various cell signalling pathways. In mammalian systems, these compounds promote and protect against inflammatory responses and cell damage [39]. In plant and microbial systems, shikonins are known to be respiratory inhibitors providing strong antifungal, antibacterial and allelopathic activity in bioassays performed under controlled conditions [23,41]. Shikonins can also induce apoptotic reactions in e.g., prostate cancer cell lines [42]. Shikonin and its derivatives (Figure 1) exhibit potent pharmaceutical activity including anti-inflammatory [23], antitumor, antibacterial, antithrombotic, antifungal, antioxidant and wound healing activities, making boraginaceous roots valuable in Far Eastern [24,37] and Western medicine [42]. Propionylshikonin was found to be an effective antiviral agent against tobacco mosaic virus [43]. Shikonins also play an important role as potent inhibitors of plant, fungal and microbial growth [23,26,41,44].

In this study we aimed to: (1) develop a platform enabling the metabolic profiling of shikonins in a large number of root samples of *E. plantagineum* and *E. vulgare*; (2) identify key secondary metabolites in root periderm using targeted and untargeted analyses; (3) evaluate the impact of plant growth stage on the biosynthesis of shikonins; and (4) investigate the importance of genotype (plant population) on shikonin biosynthesis when both species were grown under uniform controlled environmental conditions.

## 2. Results and Discussion

### 2.1. Identification of Isohexenylnaphthazarins in *Echium plantagineum* Root Periderm Extracts

**Figure 2:**
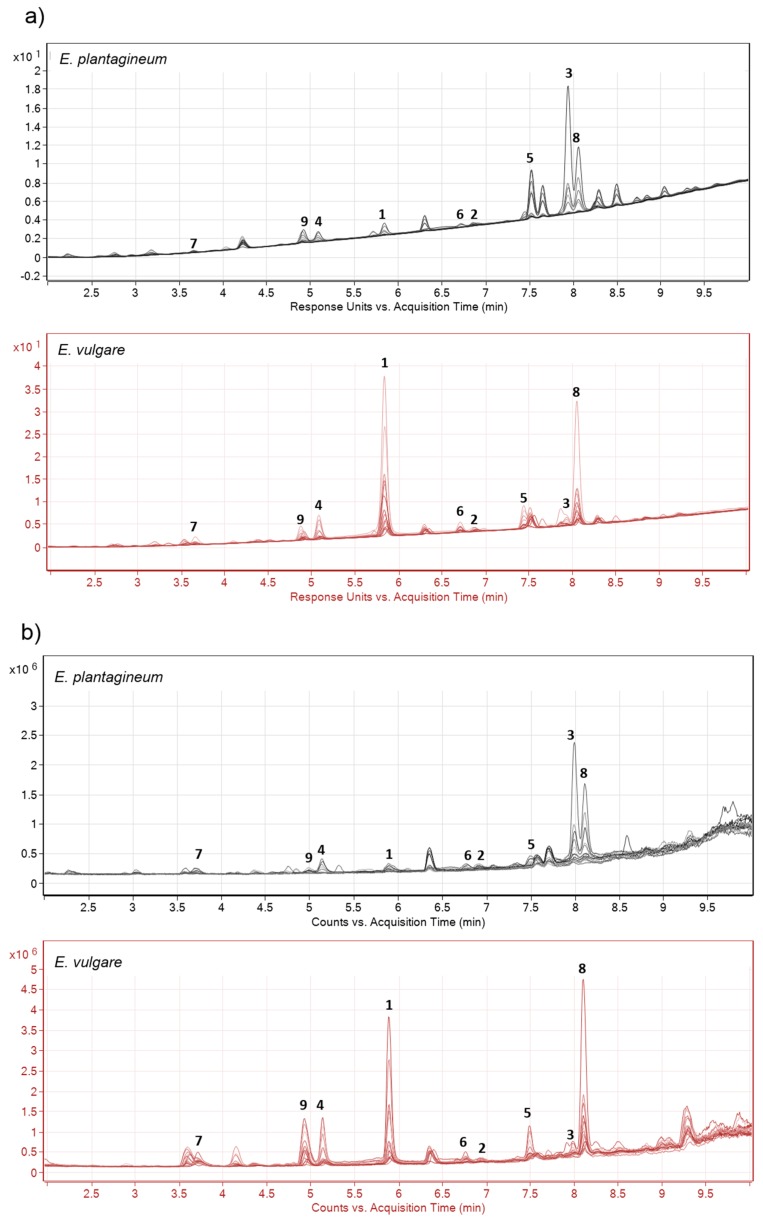
Chromatographic separation obtained from 12 selected field collected samples of *Echium plantagineum* and *E. vulgare*. (**a**) DAD chromatogram of root periderm extracts at wavelength 254 nm and (**b**) total ion chromatogram of the same extracts. Numbers **1**–**9** denote shikonins (Table 1).

Metabolic profiles of periderm extracts were generally of low complexity and contained up to 20 related and highly abundant compounds when evaluated by UHPLC Q-TOF under negative ion mode. All major chromatographic peaks in the total ion chromatogram (Figure 2b) were attributed to isohexenylnaphthazarins, indicating the presence of bioactive shikonins and consistently high activity of the biosynthetic pathway for shikonin production in the periderm. Isohexenylnaphthazarins identified in ethanolic extracts of *Echium* root periderms were classified as shikonins by accurate mass and observed fragmentation patterns using UHPLC Q-TOF and by comparison with analytical standards (Table 1) as well as previously published spectra [37]. Additional supporting information was provided by spectral evaluation of samples using diode array detection (DAD) at 254 nm (Figure 2a). Preliminary untargeted data analysis indicated the presence of additional compounds of low abundance in periderm extracts, including shikonin polymers of *m/z* 555.1650, 597.1768, 611.1915, 625.2078, 639.2242 and 655.2187; however, further experimentation is required to confirm their exact polymeric structural identity.

Shikonins are characterized by the presence of polycyclic rings with multiple quinone moieties (Figure 1); their structure is reflected in the fragmentation pattern observed which generally reveals a common fragment ion of *m/z* 270 (Figure S1). Fragmentation patterns of MS^2^ spectra (Figure S1) allowed for the positive identification of nine related isohexenylnaphthazarins (Table 1) in extracts of field-collected root periderm tissue of *E. plantagineum*, in contrast to previous methods developed in our laboratory that positively identified only four shikonins using LC/MS QQQ instrumentation [26]. Shikonin derivatives annotated in this study were generally formed by chemical modification of the side chain (Figure 1, Table 1).

Acetylshikonin (**1**), deoxyshikonin (**2**), dimethylacrylshikonin (**3**) and shikonin (**7**) (Table 1) were further identified by comparison to analytical standards and characterized based on both retention time and MS^2^ spectra. The *m/z* 270 and/or *m/z* 269 ion was specifically identified in **1** and **3** and also found in MS^2^ spectra of related shikonins for which commercial standards were not available. This distinct fragmentation pattern was also consistent with that reported by Albreht et al. [37] who presented both mass and nuclear magnetic resonance (NMR) spectra of similar isohexenylnaphthazarins in *Echium italicum* root extracts.

However, the authors did not present the accurate masses of identified compounds. Shikonins with side chain modification typically produce a product ion of *m/z* 270 or *m/z* 269, strongly suggesting they are ester derivatives of **7**, in which the ester bond is the fragmentation point. Due to differential fragmentation and variable ionization (which includes formation of free radicals) one of the most abundant fragment ions is often *m/z* 269 or *m/z* 270.

Identification and absolute quantification of shikonins is typically challenging due to both the physical and chemical properties of these compounds [45,46]. Shikonins are sensitive to heat, light, pH and oxygen, which affects their stability and may increase their degradation [45]. Compounds similar to shikonins with sufficient redox potential, including polycyclic aromatic hydrocarbons, polyenes and quinones, are known for electrolytic phenomena. In this case, shikonins in our study formed molecular radical ions by protonation/deprotonation but also by capturing or losing an electron. Shikonin derivatives, as described by Zhang et al. [46], readily form radical anions when analysed in negative ion mode using electrospray ionization source. Formation of multiple types of ions is generally dependent on solution pH, the mobile phase utilized and the ionisation source. Higher organic concentration in the mobile phase also promotes formation of [M + e]^−^ ions. In this study, each shikonin derivative was noted to form several ions including [M + e]^−^ and [M − H]^−^ (Figure S1). Spectra of compounds **4**, **6**, **7** and **9** was often dominated by the [M − H]^−^ ion; however other compounds captured electrons more frequently and formed [M + e]^−^ ions. Our results are consistent with those obtained by Zhang et al. [46] who evaluated shikonins using an LTQ Orbitrap MS and an identical mobile phase. Additional analysis by our laboratory was also performed using HPLC QQQ (Agilent 6410, Agilent, Santa Clara, CA, USA) with an electrospray ionization source and further confirmed results obtained here using UHPLC Q-TOF and those published results from an LTQ Orbitrap [46].

Multiple factors influencing ion formation render isohexenylnaphthazarins somewhat difficult to analyse and quantitate [46]. Procedures employed in this study aimed for high uniformity of sample preparation, low temperature (<25 °C) extraction and minimization of exposure to light. Good laboratory practices, optimal procedures and sample storage (−20 °C) generally minimize molecular interactions.

Key molecular features including retention time and accurate mass were noted for all molecules of interest and included for further experimentation in a personal database and library (Table 1). Since isovalerylshikonin and α-methylbutyrylshikonin showed the same *m/z* and similar fragmentation patterns, they were referred to as compound **8** and compound **9**, respectively. Although structures of both compounds were previously reported, they have identical formulae which makes their structural elucidation using UHPLC Q-TOF more challenging. Both **8** and **9** were identified in *E. italicum* root extracts, and further confirmation of structure was provided via NMR [37]. Our method allowed for fast, accurate and complete profiling of shikonins without the laborious process associated with their purification or synthesis which can also be challenging. However, absolute identification of molecular features profiled using metabolomics approaches (including shikonins) requires comparison with analytical standards.

Positive identification of **3** was obtained by comparison with the commercial standard; however, we are not able to exclude the possibility of other structurally related metabolites occurring in complex root periderm extracts considering the potential for coelution of structurally similar compounds. The DAD chromatogram of multiple overlaid samples (Figure 2a) suggests that there were likely only two compounds eluting at 8.0 ± 0.5 min. These peaks were associated with the presence of angelylshikonin, tiglylshikonin or other as yet unknown shikonins [37].

To date, UHPLC Q-TOF mass spectrometric analysis has facilitated the rapid separation and identification of nine closely related shikonins in a single analysis, without the use of multiple detection and/or structural elucidation methods. Obtained mass accuracy was highest for **4** and **5** and equalled 0.52 and 0.84 ppm, respectively (Table 1) and was similar to that obtained by Jaiswal et al. [47] using a comparable mass spectrometer; they identified over 48 alkaloids in tissues of *Aconitum* with mass accuracy approaching 1 ppm and rarely exceeding 10 ppm [47]. High mass accuracy allowed for identification of several anions formed, in comparison to less accurate mass spectrometers. The method demonstrated in this study provided direct, rapid and thorough analysis of metabolic profiles of root periderm tissues containing numerous structurally similar shikonins representative of the shikonin biosynthetic pathway encountered in *E. plantagineum* and *E. vulgare*, but did not allow for complete identification of all shikonin derivatives present.

### 2.2. Profiling of Coloured Periderm Extracts

Shikonins are red pigments, and are thought to be responsible for the red, pink or purple colouration of root periderm tissue in *Echium* spp. [24,26]. Exterior colouration of living roots varies upon harvest and ranges from bright red to tan, or brown to black. Dark colouration is mainly associated with aged roots and likely due to oxidation or polymerization of shikonins in the periderm over time [41]. Polymerization can also affect the activity and structural properties of shikonins [48]. The colour of shikonin extracts in general is dependent on the pH of the solution, and varies from red, purple and blue in acidic, neutral and alkaline pH, respectively [45].

**Figure 3:**
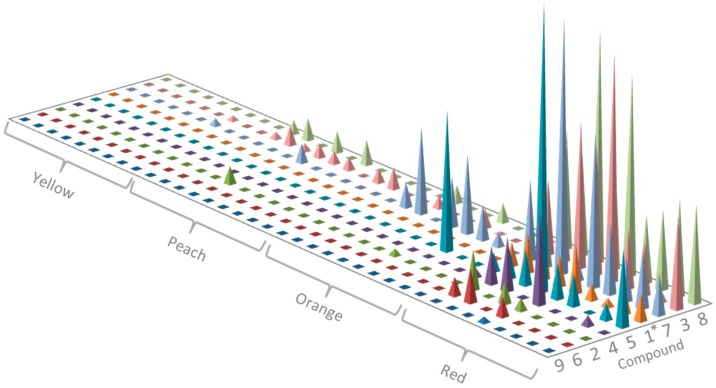
Distribution and relative abundance of nine shikonins in 36 periderm extracts separated by colouration including red, orange, peach, and yellow, with nine replicates for each colour treatment. * denotes significant positive correlation of compound abundance with extract colour.

We observed that the root periderm extracts of field-collected *E. plantagineum* samples possessed remarkable variation in colour, ranging from bright red to pink, orange to bright yellow, and pale yellow to clear. Field surveys indicated that greatest production of shikonins in *E. plantagineum* was associated with collection from northern Australian locations and sites experiencing warmer growing conditions at low elevation [26]. Thirty-six composite plant extracts obtained from a diverse collection of field-grown plants of *E. plantagineum* were ranked by colour in this experiment into four distinct colour groups and metabolic profiling was performed on a sample from each category (Figure 3 and Figure S2). Shikonins were present in red extracts; however, eight yellow to clear extracts did not contain isohexenylnaphthazarins and were excluded from statistical analysis. Peach coloured extracts generally had at least one isohexenylnaphthazarin present including **2**, **3**, **7** and **8**. Additional compounds were detected in orange-coloured extracts which were also complex, consisting of **1**, **2**, **3**, **5**, **7** and **8**. It is clear that root periderm colouration was closely related to the presence of specific isohexenylnaphthazarins, and dark orange to red colouration was associated with increasing number of isohexenylnaphthazarins in profiled extracts (Figure 3 and Figure S2). However, stepwise logistic regression analysis indicated that only the presence of compound **1**, acetylshikonin, was significantly correlated with extract colour (*p* = 0.037).

Shikonins are easily detectable by UV-Vis absorbance, and a useful model system to evaluate presence of shikonins in ethanolic extracts by colour ratings was developed, allowing ready visualisation of the products of shikonin biosynthesis and plant response to environmental stressors. Weston et al. [26] also observed more intense colouration of periderm and high total concentrations of shikonins in *E. plantagineum* collected from hot and dry areas using UV-vis absorbance, indicating shikonin biosynthesis is likely significantly upregulated under conditions of UV or drought stress. Clearly, under certain conditions in Australian soils, substantial production of shikonins occurs and is associated with increased bioactivity of extracts. The greatest phytotoxicity to annual ryegrass and/or wheat coleoptiles was observed in dark red periderm extracts of field-collected roots [8,41,44]. We also noted that concentration of acetylshikonin (**1**), was significantly higher in dark red extracts. The presence of acetylshikonin is also associated with potent antimicrobial and phytotoxic effects as assessed in crude extracts and with a purified standard [8,23,24,41,44].

Although variation in the abundance of isohexenylnaphthazarins is associated with differences in root colouration in controlled and field environments, it is not completely clear how environment influences production of shikonins in field-grown plants. Recent findings have also provided evidence that shikonins are directly released into the rhizosphere from living *Echium* roots, and can accumulate at ppm levels in *E. plantagineum*-infested soils [36,49]. Here shikonins are likely to be potent mediators of plant and microbial interactions, based on results of bioassays performed under controlled laboratory conditions. However, their role in plant invasion success is more speculative [41,49,50]. We do know that allelochemicals produced by plants can adversely impact competitors, directly or after transformation in soil, impacting physical, chemical and biological soil factors and/or affecting other trophic levels [51].

### 2.3. Time and Population Dependent Production of Isohexenylnaphthazarins

**Figure 4:**
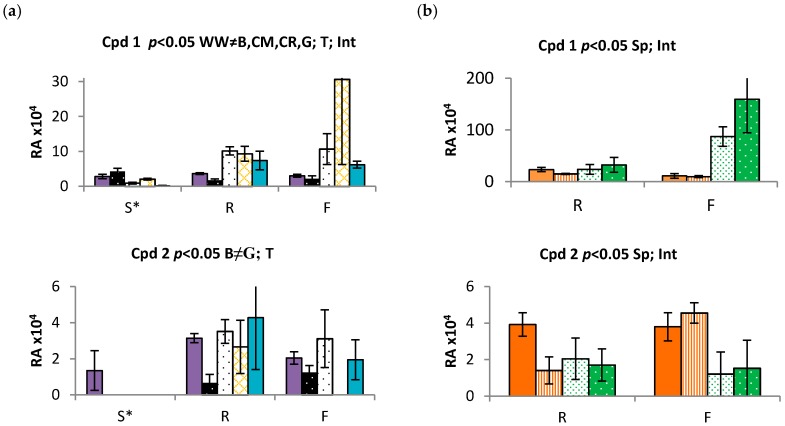

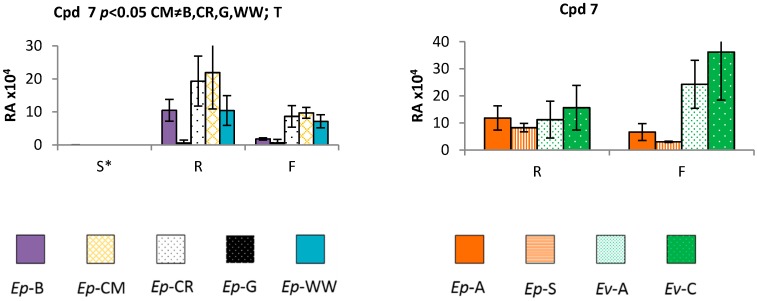
Relative abundance (RA) of **1**, **2** and **7** based on the peak area and averaged over replication in *Echium plantagineum* (*Ep*) and *E. vulgare* (*Ev*) in glasshouse controlled conditions. Error bars represent standard error of the mean (SEM). Key: (**a**) *Ep*-B-Bendigo, *Ep*-CM-Coombah, *Ep*-CR-Cobar, *Ep*-G-Grenfell, *Ep*-WW-Wagga Wagga; (**b**) *Ep*-A–Adelong, *Ep*-S-Silverton, *Ev*-A–Adaminaby, Ev-C–Cooma. Significant differences (*p* < 0.05): ≠—between populations, Sp—between species and T—between time points. Int—significant interaction, time × population. S—seedling, R—rosette, F—flowering. Data was log transformed prior to statistical analysis.

Biosynthesis of plant secondary products was shown to be related to phenological stage [52]. Increased production of secondary products at the reproductive stage was previously described in multiple genera including *Salvia*, *Hypericum*, *Thymbra* and *Satureja*, where higher levels of phenolics during flowering were involved in attracting pollinators [53]. We profiled shikonins in a controlled-environment study to evaluate the periderm of plants produced from seed of five geographically distinct Australian (Table S3) populations of *E. plantagineum*. Under identical growth conditions, the production of shikonins was significantly downregulated during the seedling stage (*p* < 0.05) and was somewhat variable among populations (Figure 4a and Figure S3a; Table S1). Shikonins were always most abundant in mature plants, and across populations were ranked in terms of total production as **8** > **3** > **7** > **1**. Acetylshikonin (**1**) was detected in all populations of 1-week-old seedlings, but was significantly less abundant in seedlings in contrast to mature plants. Acetylshikonin is one of the most bioactive isohexenylnaphthazarins in in vitro evaluation against soil bacteria and was also more active than **3** [23]. Thus, production of shikonins, including acetylshikonin**,** may be crucial in protecting seedlings and possibly mature plants against soil-borne microbes and pathogens and possibly germinating weed seedlings.

Zhu et al. [36] suggested that the root periderm forms a chemical barrier in living *Echium* roots, and contains a protective layer of isohexenylnaphthazarins, potentially enabling greater survival of seedlings. Compound **8** was also present in seedling radicle extracts in four out of five populations. Deoxyshikonin (**2**), **3** and **7** were identified only sporadically in immature seedlings, likely due to their low abundance in periderm extracts. Interestingly, **2** and **7** are precursors of other isohexenylnaphthazarins and are thought to be rapidly converted to various shikonin derivatives [24]. The positive identification of lower *m/z* isohexenylnaphthazarins in seedlings suggests that these compounds are potentially associated with the initial steps in the isohexenylnaphthazarins biosynthetic pathway.

In comparison, isohexenylnaphthazarins with greater molecular weights were found to be present in mature plants, with compounds **9** and **4** exhibiting variation depending on population and growth stage. As shikonin production is likely upregulated or induced by biotic or abiotic stimuli, and some compounds are likely transient intermediates in the shikonin biosynthetic pathway, not all shikonins were detected in all plant samples. Low abundance of **2**, deoxyshikonin, in seedling and mature plants further suggests its rapid conversion to metabolites including **1** and **7** which are highly active [23,24,41,44,54]. Dimethylacrylshikonin (**3**) was also prevalent in root extracts, however, it is not particularly bioactive [23] and may be an intermediate which is later converted to other complex metabolites. Compounds **4** and **9**, also expressed differentially among populations, may be regulated by environmental factors or genotype. 

Seedlings obtained from glasshouse culture of all populations were relatively uniform in size and appearance when grown under controlled conditions. However, despite a uniform appearance above-ground, the Coombah population produced significantly reduced levels of most shikonins of abundance (**4**, **5**, **6**, **7**, **9**). Coombah is located in a hot and dry plain at low elevation. While this population produced low levels of isohexenylnaphthazarins, plants from Cobar, another hot and dry area in NSW, were characterized by significantly enhanced production of isohexenylnaphthazarins in comparison to Coombah population (Figure 4a and Figure S3a). This finding suggests that genotype and possibly epigenetics may also play an important role in regulation of biosynthesis of isohexenylnaphthazarins and that further research into the impact of genotype on production is needed.

We also compared populations of both annual *E. plantagineum* and perennial *E. vulgare* harvested before and after floral initiation in *E. plantagineum* and at vegetative maturity in *E. vulgare*. Two populations of each species were harvested and extracted after 6–8 weeks and at 27–29 weeks, when *E. plantagineum* was fully flowering. In contrast, *E. vulgare* remained in the vegetative growth stage throughout the experiment, likely due to its perennial growth habit and lack of vernalization under controlled growth conditions [27]. Isohexenylnaphthazarins were clearly present in periderm extracts of both species, but were observed in variable concentrations (Figure 4b and Figure S3b). Highest concentrations of shikonins were observed in mature *E. vulgare*, harvested at the last sampling (Figure 4b and Figure S3b). However, similar metabolic profiles were noted in both species (Figure 4b and Figures S3b), in various populations and at both life stages, suggesting that shikonin production is highly conserved among Australian grown *Echium* species and populations. Perennial growth habit and longer periods of vegetative growth resulted in accumulation of higher concentrations of shikonins in roots of *E. vulgare*. Tight regulation of this pathway in two closely related species further suggests that the shikonin biosynthetic pathway in other *Echium* spp. may also be similar, and we note that compounds identified in *E. italicum* and *Lithospermum* spp. also suggest biosynthetic conservation of this pathway in the Boraginaceae with limited variation [24,37].

In this experiment, **9** was not found at detectable levels in *E. plantagineum* (Figure S3b) but previous experiments identified **9** in four out of five populations of this species (Figure S3a). This suggests that there are no qualitative, species-specific differences in biosynthesis of isohexenylnaphthazarins among these two *Echium* invaders. However, we did observe significant quantitative differences between *E. plantagineum* and *E. vulgare* in the abundance of **1**, **2**, **3**, and **9** (Figure 4b and Figure S3b, Table S2). In the case of **1**, **2**, and **5**, species responded differently over time with respect to production of shikonins. Significance of this interaction could be caused by variation in phenology associated with annual or perennial growth habits at the time of plant sampling or other as yet unknown factors.

The most abundant shikonins in *E. plantagineum* were consistent among experiments, with similar patterns of abundance **8** > **3** > **7** > **1**, suggesting strong conservation of the shikonin biosynthetic pathway among mature plants. Production of **1**, **4**, **5**, **6**, **9** was upregulated in *E. vulgare* at second harvest (Figure 4b and Figure S3b), while production of **3** was upregulated in *E. plantagineum*. Total abundance of isohexenylnaphthazarins at both growth stages was approximately 2.5 fold higher in the perennial *E. vulgare* when grown in a glasshouse experiment. Total isohexenylnaphthazarins in extracts were also calculated using spectrophotometric techniques and also showed a 2.5 fold higher abundance in *E. vulgare*, confirming accuracy of identification and relative quantitation using UHPLC Q-TOF MS by focusing on the most abundant ion or radical ion. Higher levels of isohexenylnaphthazarins in *E. vulgare* may be explained by upregulation of the biosynthetic pathway associated with a longer period of active growth in the vegetative stage, as *E. vulgare* plants did not flower. Due to lack of flowering, isohexenylnaphthazarin production may have been artificially elevated leading to higher concentrations of shikonins in this species after 26–29 weeks; however, field-collected samples of *E. vulgare* also showed elevated levels of total isohexenylnaphthazarins (~1.4 fold higher in comparison to field-collected *E. plantagineum*), as examined spectrophotometrically. *Echium vulgare* seeds used in this study for glasshouse experimentation were collected from naturally occurring populations at higher altitudes reflecting typical distribution in Australia (Table 2).

Highly abundant compound **3**, dimethylacrylshikonin, was less inhibitory than **1**, **2** and **7** in microbial bioassays [23], but may serve as a non-toxic and preferred storage form of isohexenylnaphthazarins in the plant and, upon demand, could potentially be converted to other bioactive forms, including polymers. Structurally complex shikonins appear to be accumulated as a result of exposure to plant stress. This suggests that the regulation of this biosynthetic pathway is more complex than previously described [55]. Shikonins provide a unique model system for study of impacts of climate change, plant genetics and plant phenotype upon gene expression, as well as regulation of plant rhizosphere interactions with other organisms.

Limited qualitative differences in shikonins between species suggest that it may be difficult to identify species-specific biomarkers in the shikonin biosynthetic pathway; however, upregulation of isohexenylnaphthazarins in flowering plants and over time suggests that regulation of biosynthesis is likely complex and is associated with both phenological and genetic factors. Cohen et al. [56] evaluated the presence and absence of isohexenylnaphthazarins and suggested this as a useful morphological feature to aid in the identification and phylogeny of Boraginaceae. However, the analysis did not focus on the presence or absence of specific shikonins [56]. Broader metabolic profiling combined with the use of powerful chemometrics software could be particularly useful for identification of differentially expressed and structurally related compounds. Additional chemotaxonomic studies in the taxonomically complex *Echium* genus would also prove valuable.

## 3. Materials and Methods

### 3.1. Chemicals and Standards

HPLC grade solvents were used in this study including acetonitrile and ethanol absolute purchased from VWR Chemicals (Tingalpa, Australia) and water ordered from Merck (Darmstadt, Germany). Formic acid, >99% purity (Sigma, Castle Hill, Australia) was used to acidify mobile phase. Chemical standards including acetylshikonin (**1**), deoxyshikonin (**2**) and dimethylacrylshikonin (**3**) were purchased from Chemfaces (Wuhan, China). Shikonin (**7**) was purchased from Biomol (Hamburg, Germany).

### 3.2. Source of Plant Material and Sample Preparation

Extracts used in the creation of personal compound database and library and in profiling coloured samples were collected in NSW, Australia, from 2013–2015. *Echium plantagineum* L. (Boraginaceae) field-collected samples were used for profiling of coloured extracts. Similarly, *E. plantagineum* and *E. vulgare* L. (Boraginaceae) roots used for compound database development and identification of isohexenylnaphthazarins were collected from the field from numerous NSW populations (Table 2). Seeds utilized in controlled conditions experiment were collected from nine locations across NSW, Australia in 2013 and 2014 (Table 2). Plants of each species were definitively identified using the DNA barcoding or sequence analysis system of key polymorphic gene regions developed by Zhu et al. [33], in comparison to herbarium voucher specimens obtained from the Australian National Herbarium in Canberra ACT.

Seeds were germinated over a week on moist filter paper in an incubator with 12 h:12 h (Light:Dark) light regime and a temperature regime of 25 °C:15 °C (Light:Dark). Following germination, seedlings were transplanted into 1.5-L pots containing 6:4 potting mix:sand. Plants were grown in the glasshouse at Charles Sturt University (Wagga Wagga, NSW, Australia) with alternating temperatures ranging from 25 °C ± 4 °C day and 17 °C ± 3 °C night. Plants were watered as needed and fertilized fortnightly with 200 mL of Aquasol soluble fertilizer (Yates, Padstow, Australia) prepared following manufacturer’s instructions. Populations of *Echium* spp. were grown in a randomised block design in three blocks for each experiment.

### 3.3. Echium plantagineum Phenological Cycle Study

Five *E. plantagineum* populations were germinated to generate 12 plants per block (Table S3). Four plants per block per population were harvested at each of three different time points: 1-week-old seedlings, 7- to 9-week-old rosettes and four 11- to 14-week-old flowering plants (flowering = over 80% with open inflorescences). Tissue of four plants derived from the same population was pooled for extraction to yield one sample per block per time point, each sample being a composite of the root periderm of four individuals to minimize plant-to-plant variation.

### 3.4. Comparative Profiling of Two Echium Species

Experimentation with *E. plantagineum* and *E. vulgare* populations was performed in order to evaluate the influence of genetics and environment upon secondary product accumulation in different species. Two populations each of *E. plantagineum* and *E. vulgare* were cultivated in a randomised block design experiment. Eight plants of each population were grown per block and later harvested at two time points: before and during flowering (Table S4) (Note: *E. vulgare* did not flower in glasshouse conditions due to its perenniating growth habit). Blocks were harvested sequentially over 3 weeks after 6–8 weeks and again after 27–29 weeks. Individuals were combined to create a composite sample of four plant root periderms as described above.

*Periderm extraction* Periderm was peeled using a sharp scalpel blade and ca. 1 g of periderm was placed in scintillation vials containing 10 mL of ethanol and placed on an orbital shaker in darkness for 14 h at 120 rpm. Composite samples consisted of equal quantities of periderm tissue from each plant (±0.02 g). Ethanolic extracts were filtered using a 0.22 µm Millipore syringe filter and stored at −20 °C until analysis.

### 3.5. UHPLC-MS Analysis

Metabolic profiling of root periderm extracts was performed using an Agilent 1290 Infinity UHPLC system equipped with quaternary pump, diode array detector (DAD), degasser, temperature controlled column and cooled autosampler compartments which was coupled to an Agilent 6530 quadrupole/time of flight (Q-TOF) mass spectrometer with Agilent Dual Jet Stream ionisation source (Agilent Technologies, Mulgrave, Australia). Extracts were separated using a C_18_ Poroshell column (2.1 mm × 100 mm, 2.7 µm particle size) (Agilent Technologies, Santa Clara, CA, USA) at 25 °C, which was connected to an SB-C_8_ guard column (2.1 mm × 12.5 mm, 5 µm particle size) (Agilent Technologies). The flow rate of the mobile phase was 0.5 mL/min. The column was equilibrated for 40 min prior to analysis. Separation was obtained using a gradient of solvent A (water, 0.1% formic acid) and solvent B (95% acetonitrile, 0.1% formic acid). The gradient began with 50% B for 1 min and reached 100% B over 7 min, continued at 100% B until 10.5 min and returned to 50% B in 0.1 min, ending with 50% B until 17 min. The DAD monitored absorbance at 254 nm. The Q-TOF was calibrated in negative ion mode with nebulizer gas set at 35 psig, capillary voltage at 3500 V and fragmentor voltage at 135 V. Nitrogen was used as the drying gas at 250 °C at a flow of 9 L/min. Data was collected in negative ion mode using extended dynamic range (2 GHz). For profiling of root extracts, scan mode was used. Based on information obtained, an auto MS-MS method was created to collect MS^2^ spectra at three different collision energies: 10, 20 and 40 eV. Fragmentation patterns were later analysed and compounds were identified based on accurate mass and product ions. 

### 3.6. Spectrophotometry

The colourimetric analysis of total isohexenylnaphthazarins was performed using a Nanodrop 2000c spectrophotometer (Thermo Scientific, Scoresby, Australia) at three wavelengths: 493 nm, 523 nm and 562 nm [26,57].

### 3.7. Data Analysis

Data obtained from UHPLC-ESI-Q-TOF analysis was primarily characterized manually in Mass Hunter Workstation Qualitative software version B06.00 (Agilent Technologies). Data were obtained using scan mode and MS-MS. Based on accurate mass and retention time (available for standards), a database was generated using averaged retention time of identified and annotated compounds and their accurate mass. Personal Compound Database and Library software (version B 04.00, Agilent Technologies) was used.

Targeted analysis of isohexenylnaphthazarins was performed using scan mode to obtain data and later analysed against personal compound database and library. The find by formula algorithm in MassHunter Workstation software (version B06.00 Qualitative Analysis, Agilent Technologies) was used to screen for compounds of interest with a retention time window of ±0.7 min. A set of standards and blanks was analysed with the samples, and the find by formula algorithm was optimized based on the results generated for standard samples. Following the analysis, chromatograms were analysed manually for presence of ions of interest to ensure annotated ions were found in the spectra. Results were later exported to compound exchange file (CEF) format. 

### 3.8. Statistical Analysis

Repeated measures analysis of variance was performed in Statistix 9 (Analytical Software, Talahassee, FL, USA) for controlled condition experiments Data on compound abundance was log-transformed prior to analyses. Standard error of the means was used to show the variability of the abundance. Stepwise logistic regression analysis on the log transformed dataset of **1**, **2**, **3**, **5** and **7** was performed to evaluate the significance of these compounds in peach, orange and red samples. Yellow was excluded from the analysis due to limited complexity and lack of compound abundance. Graphs were generated and edited in Microsoft Excel.

## 4. Conclusions

The application of a rapid UHPLC Q-TOF method for shikonin separation offered high chromatographic resolution and mass accuracy resulting in the identification of shikonins not previously noted in *Echium plantagineum* root periderm tissues. At least nine monomeric low molecular weight shikonins were detected in periderm extracts of *E. plantagineum* and *E. vulgare*. 

Metabolic profiling of root periderm extracts of invasive Australian *Echium* species indicated that the shikonin biosynthetic pathway was highly upregulated in root periderm tissues of two *Echium* invaders and both produced numerous closely related shikonins, with increasing abundance of shikonins associated with plant maturity. Shikonins were produced already at seedling stage in *E. plantagineum* and increased in abundance and diversity over time. However, total relative concentration of shikonins at maturity was higher in *E. vulgare* in comparison to *E. plantagineum*.

Shikonins were produced in a highly conserved manner in both species. This was confirmed by the lack of great variation observed in their quantitative and qualitative abundance between the species. Bioassay results suggested that shikonins play an important role in protection against soil microbiota, macrobiota and neighbouring plants in the plant rhizosphere [8,23,36,41]. The ubiquitous presence of isohexenylnaphthazarins in the root periderm of two *Echium* spp. at various phenological stages also indicated their potential significance to plant defence through the formation of a naturally occurring chemical barrier around the root [36], thereby protecting the root against invasion by soil microbiota, and likely other neighbouring plants. 

Pigmentation of the periderm was correlated to the presence of shikonins and increased in later phenological stages suggesting that the plant may become more competitive over time and should be controlled early in its development. The role of phytochemistry, plant defence and toxicity in biological invasions has not often been explored in such a great detail from a chemical perspective. However, the study of chemical, ecological and evolutionary roles of secondary products with respect to changing climates, such as that experienced in southern Australia, will be critical, particularly in relation to shifts in the spread of invasive plants over time [52].

## Figures and Tables

**Table 1:** Chromatographic and spectrometric properties of shikonins identified/annotated by LC-MS/MS. Compounds were annotated based on *m/z* of parent and product ions.

Compound No.	Compound	Formula	Calculated [M − H]	Calculated [M^−^]	Measured (*m/z*)	Δppm	Production	Used Collision Energy (CE)	Approx. RT (min)
**1**	Acetylshikonin *	C_18_H_18_O_6_	329.1031	330.1109	330.1102	2.12	270.0915	10	5.9
**2**	Deoxyshikonin *	C_16_H_16_O_4_	271.0976	272.1054	272.1048	2.21	203.0349	20	7.0
**3**	Dimethylacrylshikonin *	C_21_H_22_O_6_	369.1344	370.1422	370.1407	4.05	270.0904	20	8.0
**4**	β-hydroxyisovalerylshikonin	C_21_H_24_O_7_	387.1449	388.1528	387.1447	0.52	269.0814	10	5.2
**5**	Isobutyrylshikonin	C_20_H_22_O_6_	357.1344	358.1422	358.1425	0.84	270.0902	10	7.6
**6**	Propionylshikonin	C_19_H_20_O_6_	343.1187	344.1265	343.1169	5.25	269.0821	20	6.9
**7**	Shikonin *	C_16_H_16_O_5_	287.0925	288.1003	287.0921	1.39	218.0221	20	3.8
**8**	Compound 8 ^#^	C_21_H_24_O_6_	371.1500	372.1578	372.1582	1.08	270.0896	10	8.2
**9**	Compound 9 ^#^	C_21_H_24_O_6_	371.1500	372.1578	371.1508	2.16	269.0820	20	4.9

* Compounds analysed with analytical standards; ^#^ Structure cannot be assigned to compounds due to the same *m/z* ratio and similar fragment ions.

**Table 2:** Location and harvest date for root samples used in creation of the libraries and sources of seed used in the controlled conditions experiments.

Species	Location	Collection Year	GPS Coordinates	
			Longitude	Latitude
***E. vulgare***	Adaminaby	2013	−35.969875	148.712235
	Cooma	2014	−36.245188	149.028493
***E. plantagineum***	Adelong	2013	−35.296432	148.058302
	Bendigo	2011	−36.846232	144.181611
	Grenfell	2011	−33.918941	148.160026
	Coombah	2011	−32.980887	141.625002
	Cobar	2011	−31.524130	145.589078
	Silverton	2013	−31.879176	141.213968
	Wagga Wagga	2011	−35.054791	147.350295

## Supplementary Materials

Supplementary Materials are available online.
