# Supplementary material for: Metabolic Profiling and Identification of Shikonins in Root Periderm of Two Invasive Echium spp. Weeds in Australia
Source: Molecules. 2017 Feb 21;22(2):330. doi: 10.3390/molecules22020330 (PMC6155885; doi:10.3390/molecules22020330)
Supplement: Supplementary file 1 [file molecules-22-00330-s001.pdf]

Supplementary Materials

a) Compound 1

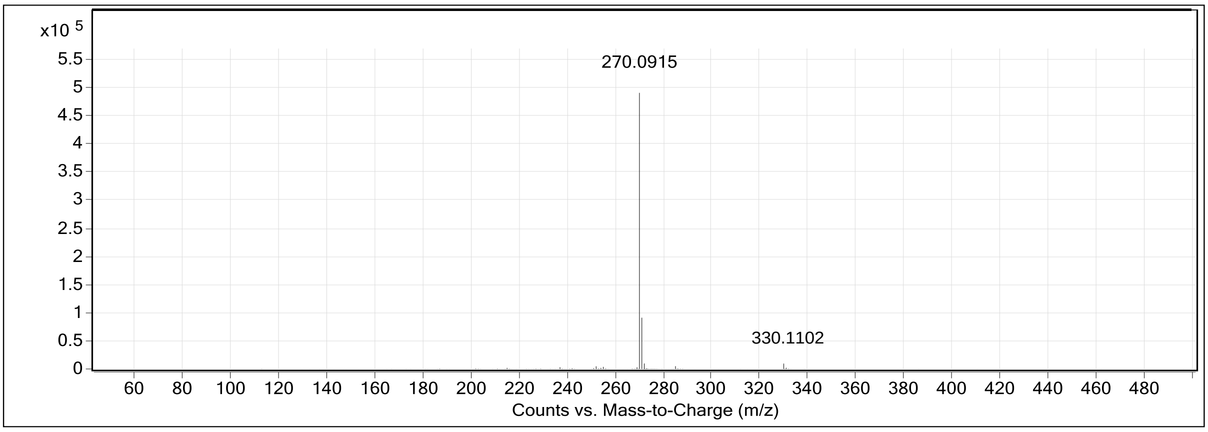

b) Compound 2

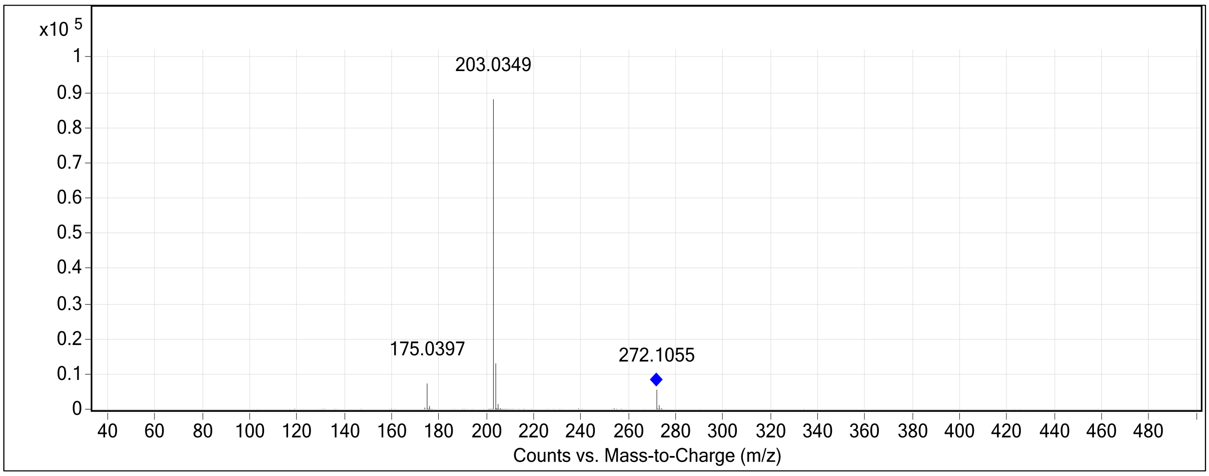

c) Compound 3

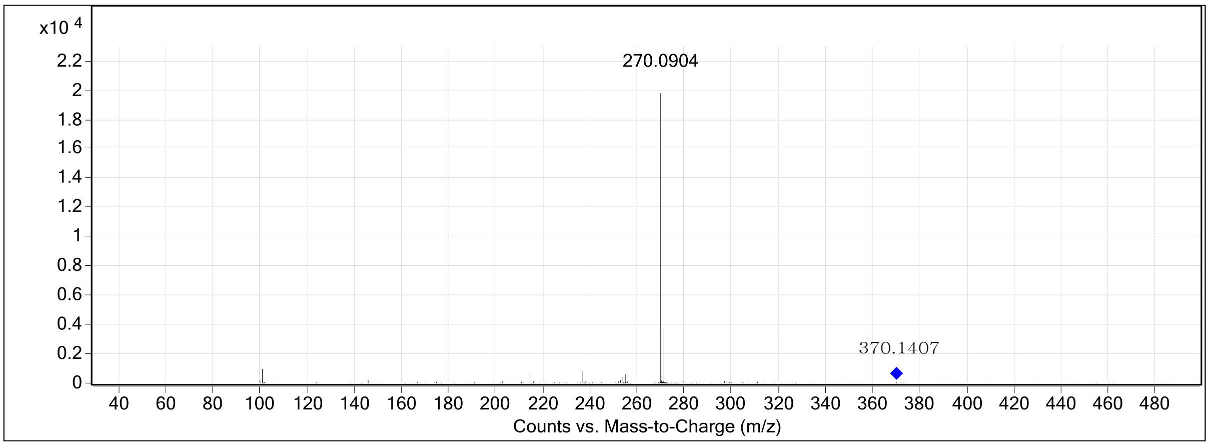

d) Compound 4

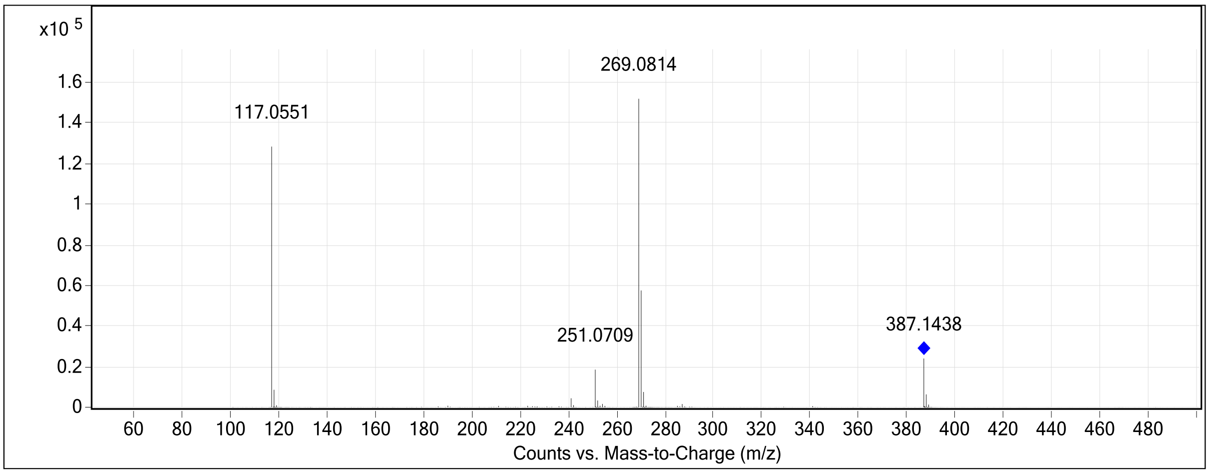

e) Compound 5

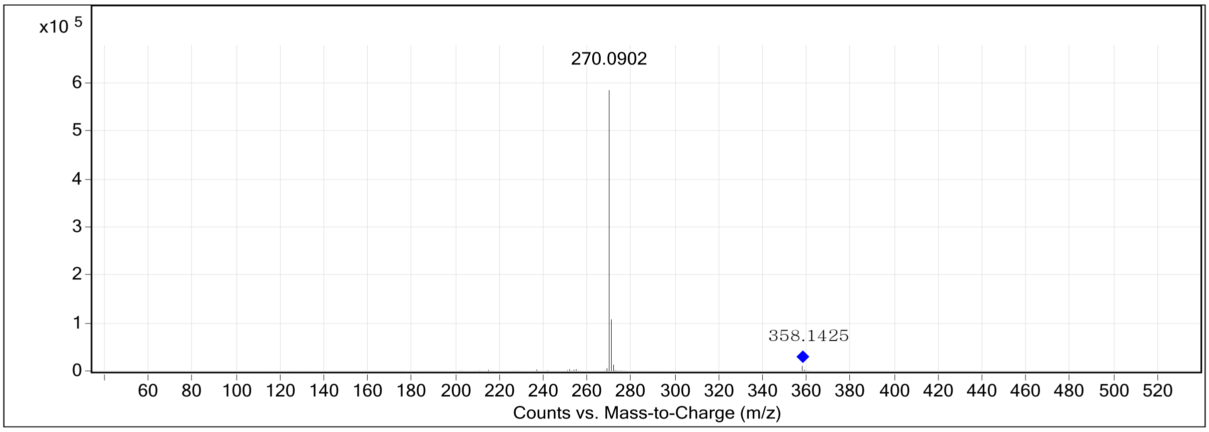

f) Compound 6

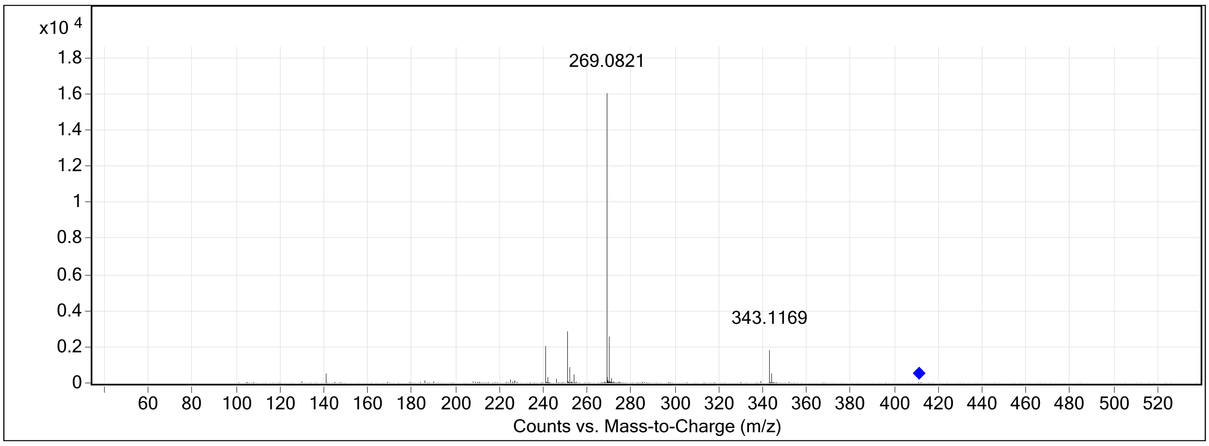

g) Compound 7

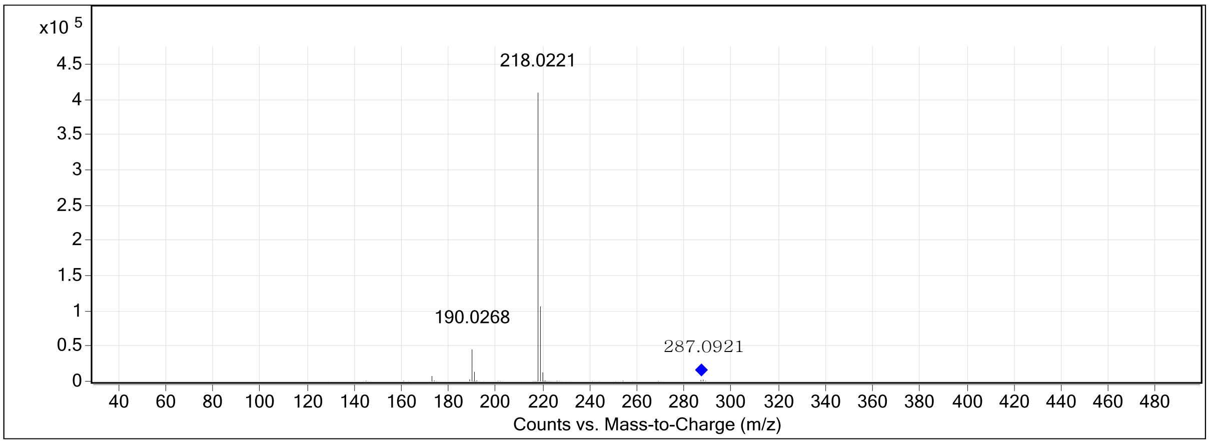

h) Compound 8

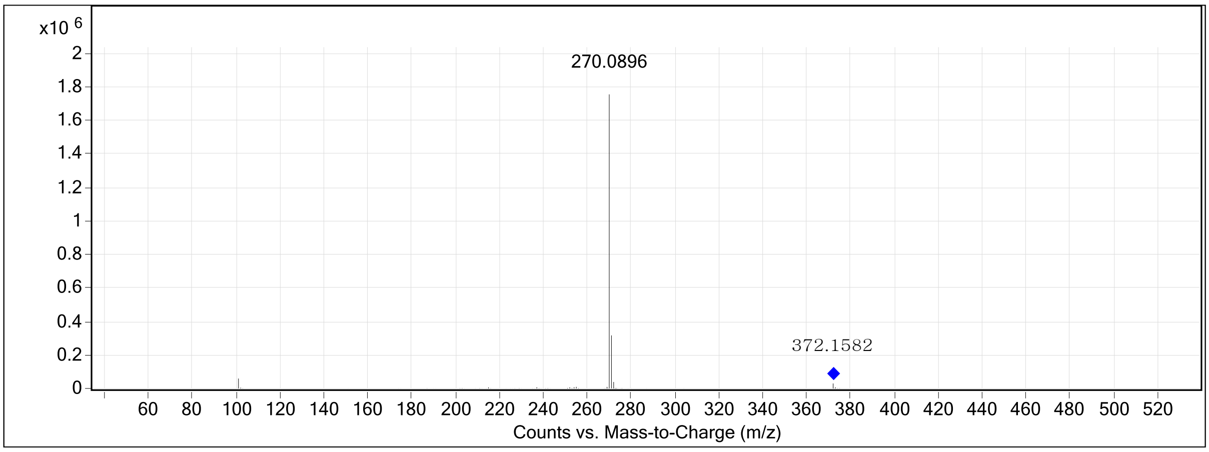

i) Compound 9

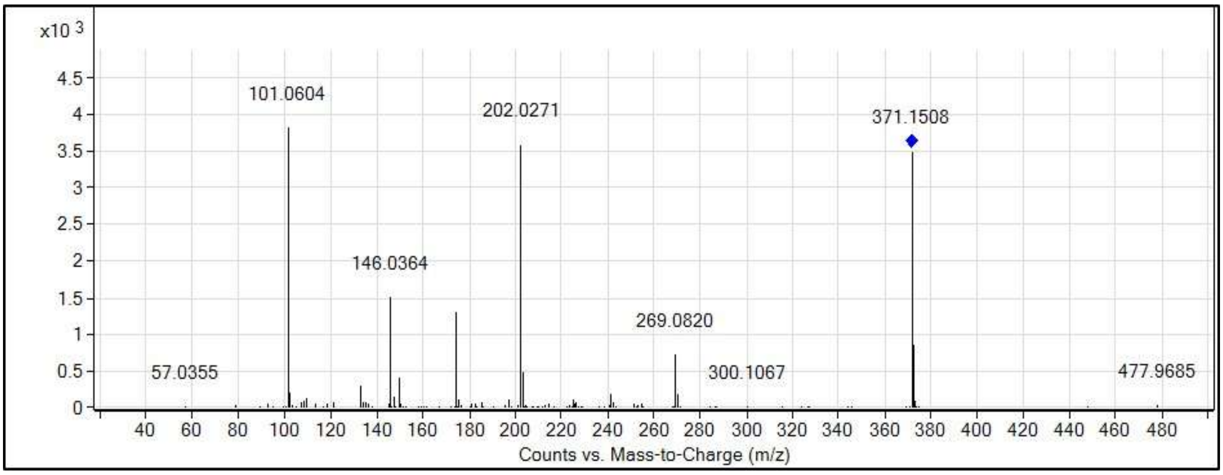

Figure 1S (a-i) – Spectral library of nine shikonins. Mass spectrum was obtained in MSMS mode using optimized collision energy (Table 1).

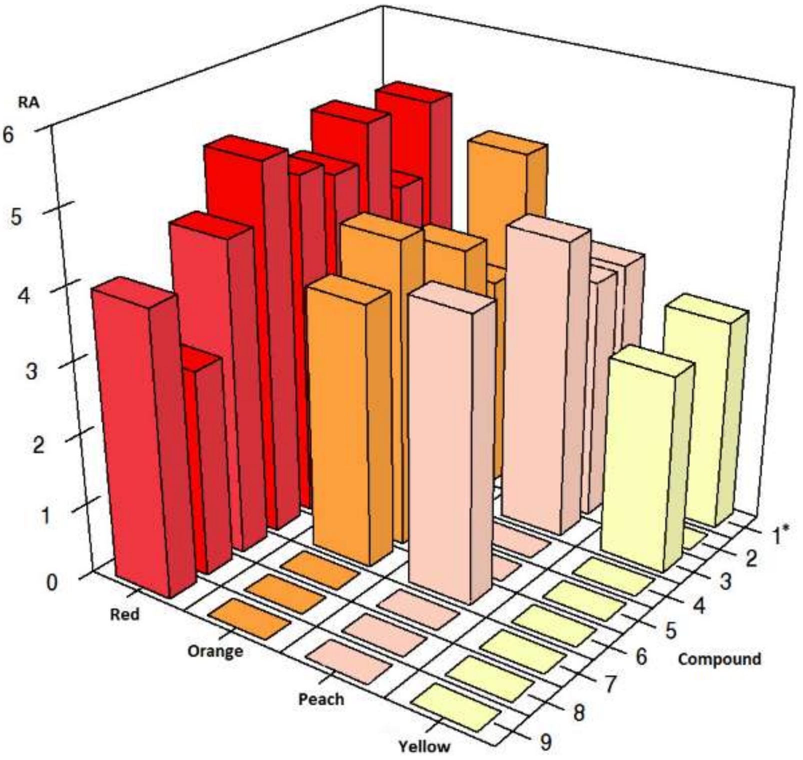

Figure 2S Distribution of nine shikonins in 36 periderm extracts separated by colouration including red, orange, peach, and yellow. Data was averaged over nine replicates for each colour treatment and log transformed. RA = relative abundance.

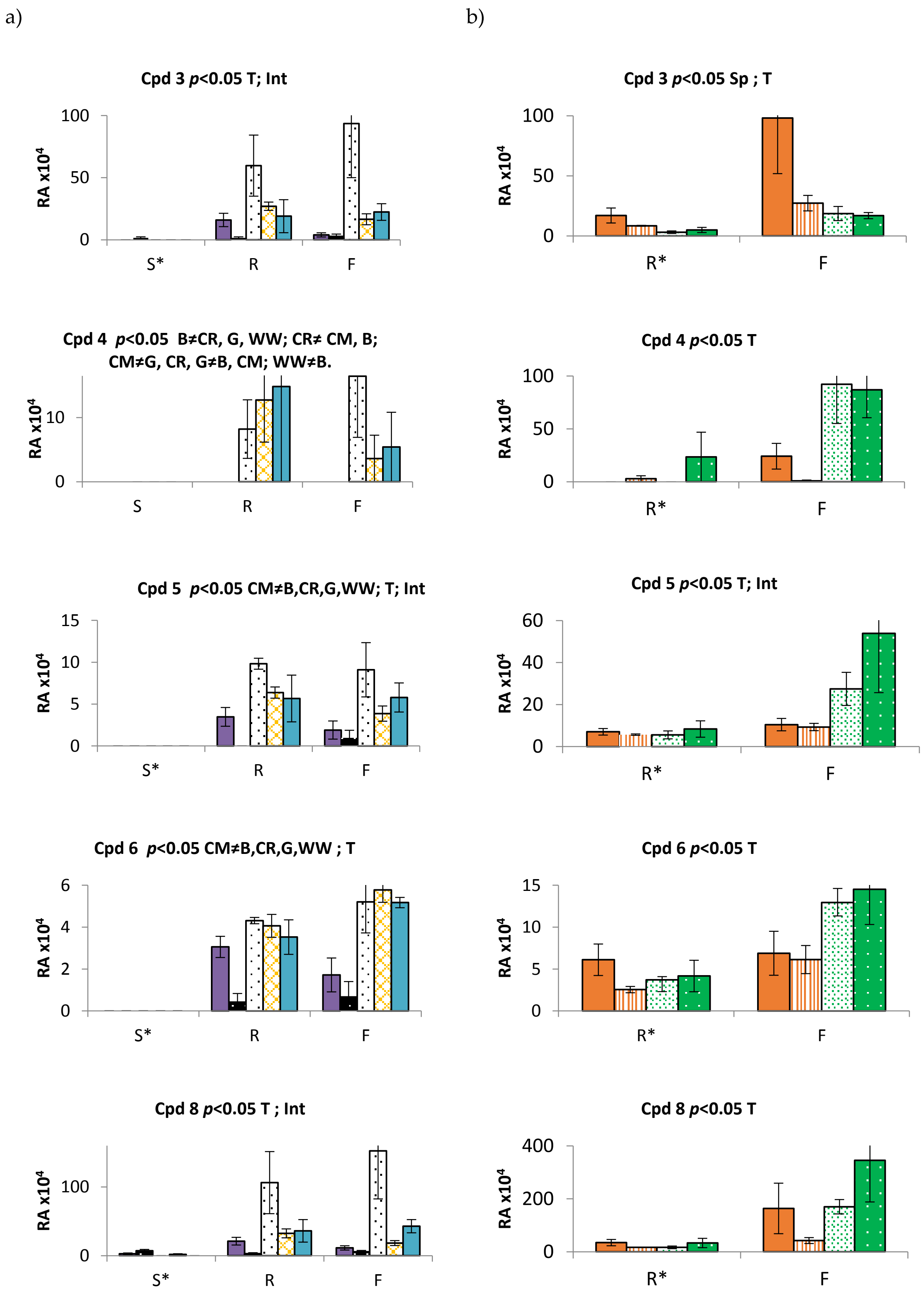
a)
b)
Cpd 3 p<0.05 T; Int
RA x10^4
100
50
0
S*
R
F
Cpd 3 p<0.05 Sp ; T
RA x10^4
100
50
0
R*
F
Cpd 4 p<0.05 B≠CR, G, WW; CR≠ CM, B; CM≠G, CR, G≠B, CM; WW≠B.
RA x10^4
10
0
S
R
F
Cpd 4 p<0.05 T
RA x10^4
100
50
0
R*
F
Cpd 5 p<0.05 CM≠B,CR,G,WW; T; Int
RA x10^4
15
10
5
0
S*
R
F
Cpd 5 p<0.05 T; Int
RA x10^4
60
40
20
0
R*
F
Cpd 6 p<0.05 CM≠B,CR,G,WW ; T
RA x10^4
6
4
2
0
S*
R
F
Cpd 6 p<0.05 T
RA x10^4
15
10
5
0
R*
F
Cpd 8 p<0.05 T ; Int
RA x10^4
100
0
S*
R
F
Cpd 8 p<0.05 T
RA x10^4
400
200
0
R*
F

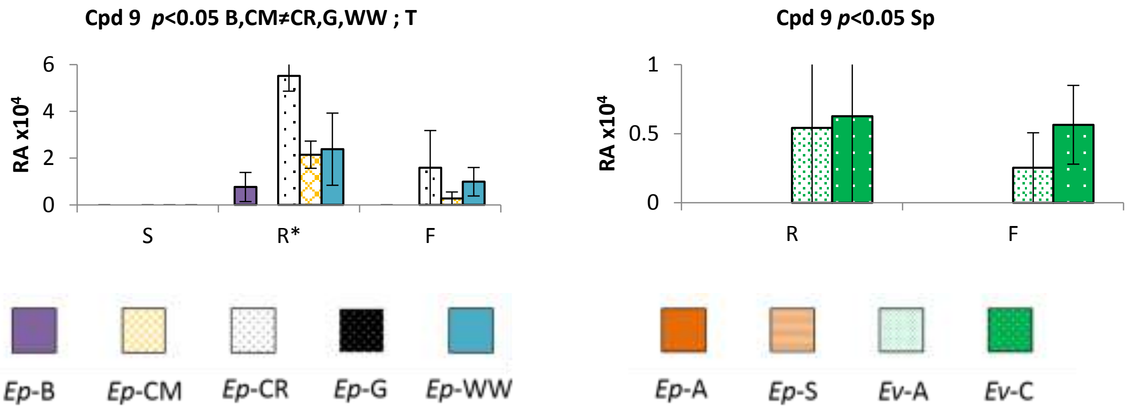

Figure 3S Relative abundance (RA) of **1**, 2 and **7** based on the peak area and averaged over replication in *Echium plantagineum* (*Ep*) and *E. vulgare* (*Ev*) in glasshouse controlled conditions. Error bars represent standard error of the mean (SEM). Key: (a) *Ep*-B-Bendigo, *Ep*-CM-Coombah, *Ep*-CR–Cobar, *Ep*-G-Grenfell, *Ep*-WW-Wagga Wagga, (b) *Ep*-A–Adelong, *Ep*-S-Silverton, *Ev*-A–Adaminaby, Ev-C–Cooma. Significant differences ($p<0.05$): '≠' - between populations, Sp – between species and T – between time points. Int – significant interaction, time × population. S- seedling, R- rosette, F-flowering. Data was log transformed proior to statistical analysis.

Table 1S. Results of repeated measures analysis of variance of relative abundance of compounds **1-9** and significance of time (growth stage), population and their interaction among five tested populations of *Echium plantagineum*. * denotes significant positive correlation of compound abundance with extract colour.

Compound	Source	df	Means square	*F* value	*p* value
**Acetylshikonin (1)**	Population	4	1.93531	5.95	0.0103
	Error Population*Replicates	10	0.32547		
	Time	2	5.03370	13.07	0.0003
	Population*Time	8	2.37977	6.18	0.0005
	Error Population*Replicates*Time	19	0.38517		
**Deoxyshikonin (2)**	Population	4	7.0020	3.24	0.0598
	Error Population*Replicates	10	2.1594		
	Time	2	40.3426	13.20	0.0003
	Population*Time	8	3.0016	0.98	0.4793
	Error Population*Replicates*Time	19	3.0568		
**Dimethylacrylshikonin (3)**	Population	4	1.0848	0.70	0.6122
	Error Population*Replicates	10	1.5601		
	Time	2	89.1036	88.10	0.0000

	Population*Time	8	3.9200	3.88	0.0073
	Error Population*Replicates*Time	19	1.0113		
**β-hydroxyisovalerylshikonin (4)**	Population	4	9.1710	7.27	0.0052
	Error Population*Replicates	10	1.2613		
	Time	2	12.4138	2.39	0.1185
	Population*Time	8	2.8478	0.55	0.8058
	Error Population*Replicates*Time	19	5.1923		
**Isobutyrylshikonin (5)**	Population	4	11.2710	12.70	0.0006
	Error Population*Replicates	10	0.8873		
	Time	2	67.0454	69.35	0.0000
	Population*Time	8	3.6352	3.76	0.0084
	Error Population*Replicates*Time	19	0.9667		
**Propionylshikonin (6)**	Population	4	6.2132	11.12	0.0011
	Error Population*Replicates	10	0.5585		
	Time	2	71.7415	48.85	0.0000
	Population*Time	8	2.0915	1.42	0.2495
	Error Population*Replicates*Time	19	1.4686		
**Shikonin (7)**	Population	4	1.328E+10	3.07	0.0686
	Error Population*Replicates	10	4.333E+09		
	Time	2	5.486E+10	10.75	0.0008
	Population*Time	8	4.934E+09	0.97	0.4896
	Error Population*Replicates*Time	19	5.105E+09		
**Compound 8**	Population	4	1.4135	1.37	0.3124
	Error Population*Replicates	10	1.0337		
	Time	2	47.8666	47.37	0.0000
	Population*Time	8	6.5141	6.45	0.0004
	Error Population*Replicates*Time	19	1.0106		
**Compound 9**	Population	4	9.1710	7.27	0.0052
	Error Population*Replicates	10	1.2613		
	Time	2	12.4138	2.39	0.1185
	Population*Time	8	2.8478	0.55	0.8058
	Error Population*Replicates*Time	19	5.1923		

Table 2S Results of repeated measures analysis of variance of relative abundance of compounds **1-9** and significance of time, species (mean of 2 populations per species) and their interaction between *Echium plantagineum* and *E. vulgare*.

Compound	Source	df	Means square	*F* value	*p* value
**Acetylshikonin (1)**	Species	1	1.94303	35.27	0.0001
	Error Species*Replicates	10	0.05508		
	Time	1	0.21809	3.02	0.1130
	Species*Time	1	1.42654	19.74	0.0012
	Error Species*Replicates*Time	10	0.07227		
**Deoxyshikonin (2)**	Species	1	22.1328	7.65	0.0199
	Error Species*Replicates	10	2.8927		
	Time	1	0.4439	0.10	0.7535
	Species*Time	1	7.9111	1.86	0.2029
	Error Species*Replicates*Time	10	4.2607		
**Dimethylacrylshikonin (3)**	Species	1	1.30689	10.40	0.0091
	Error Species*Replicates	10	0.12564		
	Time	1	2.49425	24.65	0.0006
	Species*Time	1	0.05531	0.55	0.4767
	Error Species*Replicates*Time	10	0.10117		
**β-hydroxyisovalerylshikonin (4)**	Species	1	17.9384	3.46	0.0926
	Error Species*Replicates	10	5.1898		
	Time	1	66.8822	17.52	0.0019
	Species*Time	1	14.9050	3.90	0.0764
	Error Species*Replicates*Time	10	3.8180		
**Isobutyrylshikonin (5)**	Species	1	0.33309	3.73	0.0823
	Error Species*Replicates	10	0.08931		
	Time	1	1.44927	23.48	0.0007
	Species *Time	1	0.58393	9.46	0.0117
	Error Species*Replicates*Time	10	0.06173		
**Propionylshikonin (6)**	Species	1	0.08855	0.97	0.3478
	Error Species*Replicates	10	0.09125		
	Time	1	1.08504	10.27	0.0094
	Species*Time	1	0.35474	3.36	0.0968
	Error Species*Replicates*Time	10	0.10566		
**Shikonin (7)**	Population	1	0.00137	0.00	0.9718
	Error Species*Replicates	10	1.04371		

	Time	1	0.74312	0.54	0.4803
	Species*Time	1	2.94288	2.13	0.1753
	Error Species*Replicates*Time	10	1.38267		
**Compound 8**	Population	1	0.28700	2.56	0.1407
	Error Species*Replicates	10	0.11214		
	Time	1	3.30200	21.82	0.0009
	Species*Time	1	0.56719	3.75	0.0816
	Error Species*Replicates*Time	10	0.15131		
**Compound 9**	Species	1	17.0331	7.33	0.0220
	Error Species*Replicates	10	2.3223		
	Time	1	0.4395	0.19	0.6761
	Species*Time	1	0.4395	0.19	0.6761
	Error Species*Replicates*Time	10	2.3729		

Table 3S *Echium plantagineum* samples collected from glasshouse experiment in the study of phenological cycle for each population including: Bendigo, Grenfell, Coombah, Cobar, Silverton, and Wagga Wagga.

Plant age [weeks]	Phenological stage	Block
1	S	1
1	S	2
1	S	3
7	R	1
8	R	2
9	R	3
12	F	1
13	F	2
14	F	3

Table 4S Plant samples obtained from controlled conditions experiment using two populations of *E. plantagineum* and two populations of *E. vulgare*, harvested at two time points. Each sample was a composite of four plant extracts. Key: F-flowering stage; R-rosette stage. Note: at both harvest dates, *E. vulgare* was at rosette stage.

Species	Location	Plant age [weeks]	Phenological state	Block
E. plantagineum	Adelong 1	6	R	1
	Adelong 1	27	F	1
	Adelong 2	7	R	2
	Adelong 2	28	F	2
	Adelong 3	8	R	3
	Adelong 3	29	F	3
	Silverton 1	6	R	1
	Silverton 1	27	F	1
	Silverton 2	7	R	2
	Silverton 2	28	F	2
	Silverton 3	8	R	3
	Silverton 3	29	F	3
E. vulgare	Adaminaby 1	6	R	1
	Adaminaby 1	27	R	1
	Adaminaby 2	7	R	2
	Adaminaby 2	28	R	2
	Adaminaby 3	8	R	3
	Adaminaby 3	29	R	3
	Cooma 1	6	R	1
	Cooma 1	27	R	1
	Cooma 2	7	R	2
	Cooma 2	28	R	2
	Cooma 3	8	R	3
	Cooma 3	29	R	3
